# Heat stress induced testicular impairment is related to orchitis and complement activation in Rongchang boars

**DOI:** 10.1186/s40104-025-01296-5

**Published:** 2025-12-17

**Authors:** Xiangyuan Ma, Wenxue Shen, Junhao Ni, Xihao Luo, Lianqiang Che, Bin Feng, Lun Hua, Yong Zhuo, Zhengfeng Fang, Shengyu Xu, Jian Li, Xuemei Jiang, Yan Lin, De Wu

**Affiliations:** 1 Institution of Animal Nutrition, Sichuan Agricultural University, Chengdu, People’s Republic of China; 2 Key Laboratory for Animal Disease-Resistant Nutrition of the Ministry of Education of China, Sichuan Agricultural University, Chengdu, People’s Republic of China; 3 Key Laboratory of Animal Disease-Resistant Nutrition of Sichuan Province, Sichuan Agricultural University, 211 Huimin Road, Wenjiang District, Chengdu, People’s Republic of China

**Keywords:** Boar, Complement, Heat stress, Macrophage, Orchitis, Semen quality, Testis

## Abstract

**Background:**

Heat stress (HS) is posing as a tremendous threat to the swine industry, due to the thermos-sensitive gonads of boars. Testes are immune-privileged organs in which spermatogenesis needs to remain undisturbed, whereas immune cells are thermo-sensitive, especially macrophages, which are the most abundant testicular immune cells. Our study aimed to unveil the underlying immune responses and assess their consequences on the semen quality of boars under HS. The results will aid in addressing environmental temperature-related seasonal infertility and in selecting the best boar for use in artificial insemination.

**Methods:**

The 3-week experiment assigned 26 8-week-old Rongchang male pigs into thermal neutral pair-feed (TN-PF) and HS groups. During the last 2 weeks, which served as the HS period, the HS group was subjected to 14-day 35 ± 1 °C, while the TN-PF group was kept at 26 ± 1 °C. Pig gonad tissues were sampled at the end of HS period for assessments and measurements.

**Results:**

Our findings confirmed HS-related reactions such as elevated respiration rate (*P* < 0.05) and elevated heat shock protein 60 (HSP60; *P* < 0.05) and heat shock protein 90 (HSP90; *P* < 0.05) expression levels. Sperm motility (*P* = 0.06) and progressive sperms (*P* = 0.08) were decreased under HS as was a significant reduction in average straight-line velocity (VSL; *P* < 0.05). Additionally, total abnormality levels increased (*P* < 0.05). Fibrosis, caspase-3 expression, and accumulations of tumor necrosis factor-α (TNF-α; *P* < 0.05) and interleukin-1β (IL-1β; *P* < 0.05), along with an elevated macrophage composition (*P* < 0.05) characterized the orchitis under HS. Single cell RNA sequencing (scRNA-seq) revealed fluctuations in engulfing and inflammatory signals in testicular macrophages (TMs). In particular, the complement cascade was promoted by CD163^+^ macrophages, resulting in membrane attack complex (C5b-9) assembly (*P* < 0.05). Linear regressions further revealed a negative correlation between C5b-9 and sperm motility (*P* < 0.05), as well as near-negative correlations between the C5b-9 and both progressive motility (*P* = 0.08) and VSL (*P* = 0.06).

**Conclusions:**

Our findings highlighted the relationship between HS, the onset of orchitis, and the activation of the complement system, all of which decreased the boar semen quality.

**Supplementary Information:**

The online version contains supplementary material available at 10.1186/s40104-025-01296-5.

## Introduction

Boars are fundamental to the modern swine industry due to their broad impact on sows and resulting offspring owing to the universal use of artificial insemination techniques. However, during the global climate change, HS is becoming an unignorable threat to boars [1]. After only 72 h under conditions of 33 °C and 50% relative humidity (RH), boar semen quality decreased and remained poor for the next 2 months [2]. The continuous decrease in sperm viability and increase in the sperm apoptosis rate has been reported after 2 weeks of HS [3]. More importantly, Cameron and colleagues have proven that semen from heat stressed boars directly impacts the reproductive performances of sows by decreasing the pregnancy rate, normal embryo numbers, and embryo survival rate. These each directly decrease the output of piglets [4].

Testes, as the only site for spermatogenesis, are key to semen quality. Although the thermo-regulating mechanisms of the scrotum attempt to maintain the testes at the appropriate temperature for spermatogenesis, the scrotal temperature will be affected by HS because it is located outside the body core [5]. Scrotal temperature disruption like HS, varicocele and cryptorchidism have been reported to severely dampen male fertility, yet their mechanisms have not been fully elucidated [6–10]. The testes are regarded as an immune-privileged organ to maintain tolerance to self-antigens and spermatogenesis undisturbed [11, 12]. Unfortunately, heat is naturally related to immune activity [13]. For example, the exposure of macrophage, the most abundant testicular immune cell, to high temperatures directly triggers the expression of inflammatory cytokines and increases macrophage engulfment activity levels [14–16]. Among the inflammatory cytokines produced by macrophages, the complement C1Q and its down-stream C5b-9 lead to membrane damage. They are responsible for testicular impairment, and the connection between C1Q and HS has already been shown in other animal models [17–20].

Finally, we hypothesized that HS would lead to testicular inflammation characterized by a promoted complement cascade, which directly impact semen quality of boars. With assistance of omics technology, we devoted to clarify the effect of HS on the immunity of testis and its possible connection to HS induced reproductivity changes in Rongchang boars after 14 d at 33 °C.

## Materials and methods

### Animals and experimental design

In total, 26 8-week-old male Rongchang pigs were selected and individually housed in stainless-steel metabolism cages at 26 ± 1 °C for adaption. Boars had free access to water, and feed was supplied four times per day at 8:00, 12:00, 16:00, and 20:00. The feed was formulated to meet the nutrient requirements of swine, providing 3.15 Mcal/kg metabolizable energy and 15% crude protein. After 3 weeks of adaption and 1 week prior to the HS treatment, 25 80-day-old pigs were randomly assigned to the HS or TN-PF group. One pig was excluded because of cryptorchidism. The experiment was carried out with a one-way treatment design and a randomized complete block experimental design with one pig representing one experiment unit. In addition to the house’s ventilation system, warm air blowers and heat lamps were used in controlling the room temperature at 35 ± 1 °C for 14 d for the HS group, whereas the room temperature of TN-PF group was maintained at 26 ± 1 °C.

### HS and temperature measurement

Room temperature and RH were recorded every hour by the auto-thermometer (Renke, Jinan, China) during the whole experimental period, and the temperature-humidity index (THI) was calculated based on THI = (1.8 × T + 32) – (0.55 – 0.55 × RH × 0.01) × (1.8 × T – 26). Scrotal temperature was measured using an infrared thermometer (Hikvision, Hangzhou, China), and respiration rate was measured manually.

### Sample collection

At the end of the experiment, pigs were anaesthetized by 2 mL intramuscular injected Zoletil™50 (2 mg/kg Tiletamine and 2 mg/kg Zolazepam; Virbac, Nice, France) before cutting along the ventral midline to expose the abdominal cavity. Afterwards, blood from spermatic and portal veins was collected using vacuum blood collection tubes containing heparin sodium and disposable blood collection needles. The plasma was separated and stored at −20 °C after centrifugation at 3,000 × *g* for 15 min. The pigs were then slaughtered unconscious before testes and epididymides were acquired. A part of each testis and intact epididymis was subsequently sent to a laboratory within 5 min for further assays, whereas the remainders were quickly frozen using liquid nitrogen.

### Semen quality analysis

After epididymides were transported to the laboratory, the cauda epididymides were separated and cut into small piece. The pieces were placed in a tube containing 10 mL of semen extender. After being placed in a 37 °C water bath for 15 min, supernatants were collected and fivefold diluted before analyzing using a computer-assisted sperm analysis system (Minitube, Tiefenbach, German). The supernatants were also used in the preparation of sperm slides. Briefly, 20 μL of the supernatant was pipetted onto a slide and dispersed. Each slide was next air dried and fixed using Immunol Staining Fix Solution (Beyotime, Shanghai, China) for 15 min. Then, it was washed using ultrapure water and air dried before staining with Crystal Violet-Gentian Violet Stain Solution (Beyotime, Shanghai, China) for 15 min. Finally, the prepared slide was washed and dried again before being placed in sealed bags for later morphological observation.

### Flow cytometry analysis

Testis tissues were first sliced and incubated in Hanks Balanced Salt Solution (Solarbio, Beijing, China) containing 1 mg/mL collagenase I (Sigma, St. Louis, Missouri, USA) and 100 U/mL DNase I (Solarbio, Beijing, China) at 37 °C water bath for 15 min. Then, 1 mL fetal bovine serum (Clark, Richmond, Virginia, USA) was added to the supernatant to stop digestion. Afterwards, the suspension was filtered through a 70-µm cell strainer (Nest, Wuxi, China) and centrifuged at 350 × *g* for 5 min at 4 °C. After supernatant removal, cells were resuspended in Dulbecco's Phosphate-Buffered Saline (Gibco, Carlsbad, California, USA) containing fixable viability stain 780 (BD, Franklin Lakes, New Jersey, USA) at 4 °C for 30 min. The suspension was subsequently centrifuged at 350 × *g* for 5 min at 4 °C before removing the supernatant. The remaining cells were resuspended in 100 μL Stain Buffer (BD, Franklin Lakes, New Jersey, USA) containing mouse anti-pig CD45: FITC (Bio-Rad, Hercules, California, USA), mouse anti-pig CD163: RPE (Bio-Rad, Hercules, California, USA), and BV421 rat anti-CD11b (BD, Franklin Lakes, New Jersey, USA) at 4 °C for 30 min. Each suspension was centrifuged at 350 × *g* for 5 min at 4 °C to remove the supernatant. Afterwards, 250 μL of Cytofix (BD, Franklin Lakes, New Jersey, USA) was used to fix the cells at 4 °C for 20 min. The suspension was then centrifuged at 350 × *g* for 5 min at 4 °C before removing the supernatant. Cells were later resuspended in 500 μL of Cytoperm (BD, Franklin Lakes, New Jersey, USA) to permeate the cell membrane for 5 min. After centrifugation at 350 × *g* for 5 min at 4 °C and the disposal of Cytoperm, 100 μL of Cytoperm containing mouse anti-pig macrophages: Alexa Fluor 647 (Bio-Rad, Hercules, California, USA) was added to resuspend the cells at 4 °C for 40 min. Lastly, each cell suspension was centrifuged at 350 × *g* and 4 °C to remove the supernatant and washed once with 100 μL Stain Buffer. Then, 350 μL of Stain Buffer was used to resuspend the cells for analysis using a BD Verse flow cytometer (BD, Franklin Lakes, New Jersey, USA).

### ELISA assays

ELISA assays were carried out in accordance with the manufacturer’s instructions and included HSP60 (BIM, San Francisco, California, USA), HSP90 (BIM, San Francisco, California, USA), 8-hydroxydeoxyguanosine (8-OHG; BIM, San Francisco, California, USA), testosterone (BIM, San Francisco, California, USA), IL-1β (BIM, San Francisco, California, USA), TNF-α (R&D, Minneapolis, Minnesota, USA), C1Q (BIM, San Francisco, California, USA), C3 (BIM, San Francisco, California, USA), C5b-9 (BIM, San Francisco, California, USA), and IgG (BIM, San Francisco, California, USA).

### Histological analysis

Masson slides were prepared by Hubei BIOSSCI Biotech Co., Ltd. Tissue slides were immersed in clearer for 10 min. This step was repeated two times, with excess liquid gently shaken off between steps. Tissue slides were immersed in progressively diluted ethanol solutions. The dehydrated tissue slides were immersed in Bouin’s solution or Zenker’s solution overnight and then rinsed with running water. Slides were stained with hematoxylin solution or iron hematoxylin for 5–10 min and washed gently with running water. Slides were differentiated using 0.8%–1.0% hydrochloric acid alcohol and washed with running water for several minutes. Slides were then treated with lithium carbonate solution, washed with running water and ultimately immersed in distilled water to rehydrate the tissue. They were then subjected to rinses in decreasing ethanol concentrations as follows: absolute ethanol for 5 min, 95% ethanol for 5 min, 85% ethanol for 5 min, and 75% ethanol for 5 min. They were then rinsed with distilled water for 1 min. Slides were stained with ponceau acid fuchsin solution for 5–10 min and washed with running water. Slides were treated with phosphomolybdic acid solution for 5 min and then stained with aniline blue solution for 5 min without washing. Slides were treated with 1% glacial acetic acid for 1 min and dehydrated several times with 95% alcohol. The tissue slides were dehydrated with absolute alcohol, made transparent with xylene, and then mounted using neutral balsam.

Immunohistochemistry slides were prepared by Hubei BIOSSCI Biotech Co., Ltd. Briefly, deparaffinization and rehydration were carried out. Then, EDTA antigen retrieval solution was used for antigen retrieval. Afterwards, each slide was immersed in 3% H_2_O_2_ to block the innate peroxidase. Then, each slide was blocked using 10% rabbit serum solution. The slides were subsequently stained using a rabbit anti-caspase-3 (Cell Signaling Technology, Danvers, Massachusetts, USA) antibody solution at 1:200. Finally, the immunohistochemistry slides were stained using a goat anti-rabbit IgG-HRP secondary antibody (Abcam, Cambridge, UK) at 1:2,000 before being subject to the 3,3'-diaminobenzidine (Maxim, Fuzhou, China) chromogenic reaction.

Immunofluorescence slides were prepared by Hubei BIOSSCI Biotech Co., Ltd. Briefly, deparaffinization and rehydration were carried out. Then, EDTA antigen retrieval solution was used for antigen retrieval. Afterwards, each slide was blocked using 10% goat serum solution. The slides were subsequently stained using a goat anti-pig IgG antibody (Thermofisher, Waltham, Massachusetts, USA) solution at 1:20.

### Non-targeted metabolomics

Non-targeted metabolomics was performed by LC-Bio Technology Co., Ltd. (Hangzhou, China). The collected spermatic vein plasma was thawed on ice, and metabolites were extracted with 80% methanol buffer. Briefly, 100 μL of each sample was extracted using 400 μL of precooled methanol. The extraction mixture was then stored for 30 min at −20 °C. After centrifugation at 20,000 × *g* for 15 min, the supernatants were transferred to new tubes and vacuum dried. The samples were then redissolved with 100 μL 80% methanol and stored at −80 °C prior to the Liquid Chromatograph-Mass Spectrometer analysis.

### Transcriptomics

RNA-sequencing (RNA-seq) was performed by LC-Bio Technology Co., Ltd. Briefly, total RNAs of testes were isolated and purified using TRIzol reagent (Invitrogen, Carlsbad, California, USA) following the manufacturer's procedure before they were reverse-transcribed to cDNAs using SuperScript™ II Reverse Transcriptase (Invitrogen, Carlsbad, California, USA). The cDNA samples were next processed and sequenced on an Illumina Novaseq™ 6000 following the vendor's recommended protocol.

For the RNA-seq data analysis, we used HISAT2 (https://ccb.jhu.edu/software/hisat2) to map reads to the reference genome of Sus_scrofa.Sscrofa11.1. (http://ftp.ensembl.org/pub/release-107/fasta/sus_scrofa/dna/). The mapped reads of each sample were assembled using StringTie (https://ccb.jhu.edu/software/stringtie) with default parameters. StringTie was subsequently used to determine expression levels of mRNAs by calculating FPKM, as follows: FPKM = [total_exon_fragments/mapped_reads (millions) × exon_length (kb)]. The differentially expressed mRNAs meeting the criterium fold change (FC) > 2 or FC < 0.5 were selected, and parametric F-tests were performed comparing nested linear models (*P* < 0.05) using R package edgeR (https://bioconductor.org/packages/release/bioc/html/edgeR.html). Afterwards, Kyoto Encyclopedia of Genes and Genomes (KEGG) and Gene Ontology (GO) enrichments were assessed using the differentially expressed genes (DEGs).

### Single cell transcriptomics

scRNA-seq was performed by LC-Bio Technology Co., Ltd. First, testis tissues were lysed to form cell suspensions, and then, mouse anti-pig CD45: FITC (Bio-Rad, Hercules, California, USA) and FACS sorting were applied to prepare testicular CD45^+^ cells that were used in the later generation of nanoliter-scale gel bead-in-emulsions (Fig. S3A). After releasing mRNAs from cells in the emulsions, cDNA was produced by reverse transcription and was later amplified by PCR to construct single-cell libraries. These libraries were sequenced on a NextSeq 500 sequencing system. The scRNA-seq quantification and quality control were performed using Cell Ranger (6.0). Then, R package Seurat was used to filter abnormal data and to perform preliminary clustering and visualization. Afterwards, cells were identified by repeatedly comparing the cell markers of clusters to the Cell Taxonomy database (https://ngdc.cncb.ac.cn/celltaxonomy/) before being labeled. Using a Bimod Likelihood-ratio test with criteria *P* < 0.01 and log_2_FC ≥ 0.26, DEGs were revealed between groups, and pathway enrichments were performed using the DEGs. Re-clustering was performed based on the identified clusters, and DEG identification and KEGG enrichment were carried out.

### Conventional statistical analysis

Data was analyzed by Mixed Procedure in SAS (9.4), using the following model:

$$\text{Y }=\text{ mean }+\text{ treatment }+\text{ block }+\text{ error},$$

where mean = overall means, treatment = treatment effect, block = random effect = maternal origin, and error = residual. Samples were identified as outliers if the standard residual (residual/standard deviation) was larger than 2.5. Normality and homogeneity of variances were evaluated using Shapiro-Wilk and Levene tests, respectively. Data were transformed if they did not fit a normal distribution, and an unequal variance analysis by Mixed Procedure was applied if variances were significantly unequal. Data requiring repeated measurements was analyzed using Mixed Procedure. Grouping letters were assigned by the SAS Macro program. All data were presented as mean ± standard error of the mean (SEM). Additionally, *P* < 0.05 was considered statistically significant and 0.05 < *P* < 0.10 was regarded as showing a tendency. Linear regression and correlation analysis was performed using OmicStudio tools at https://www.omicstudio.cn/tool.

## Results

### Heat stress and semen quality of boars

**Fig. 1:**
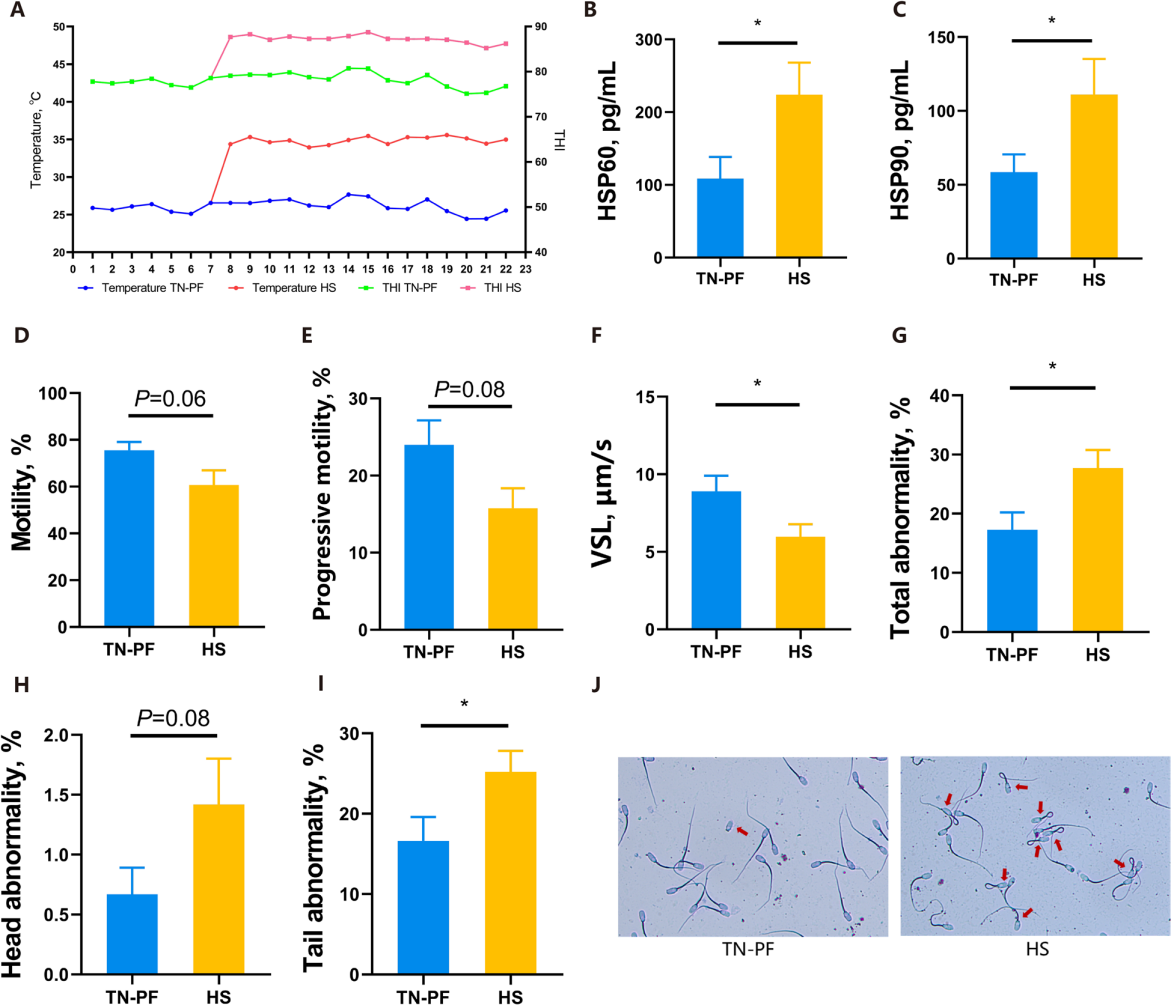
HS reactions and semen quality of boars. **A** Room temperature and THI during the experiment. **B** and** C** Quantification of HSP60 and HSP90 in plasma isolated from the spermatic vein. **D–I** Sperm motility (**D**), progressive motility (**E**), VSL (**F**), total abnormality ratio (**G**), head abnormality ratio (**H**), and tail abnormality ratio (**I**). **J** Representative photos of abnormal sperm

As shown in Fig. 1A, the THI remained above 86 and the room temperature was kept at 35 ± 1 °C during the HS period. There were typical HS reactions in boars, with both their respiration rate (RR) and scrotal temperatures being elevated (*P* < 0.01; Fig. S1A–C). Additionally, plasma HSP60 and HSP90 isolated from spermatic veins of HS boars were significantly higher compared with those of the TN-PF group (*P* < 0.05; Fig. 1B and C). Therefore, the HS reaction was confirmed to occur in both the boars and their scrotums.

The HS decreased the semen quality of boars. There were reduced percentages of motile sperms and progressive motile sperms under HS conditions (*P* = 0.06 and *P* = 0.08, respectively; Fig. 1D and E). In addition, there was significant reduction in VSL (*P* < 0.05; Fig. 1F). Moreover, there was an increased tendency toward sperm head abnormality (*P* = 0.08; Fig. 1H), as well as significant elevations in tail abnormality and total abnormality (*P* < 0.05; Fig. 1G, I and J). Together, our findings revealed decreased porcine semen quality under HS conditions.

### HS facilitated orchitis and testicular impairment

**Fig. 2:**
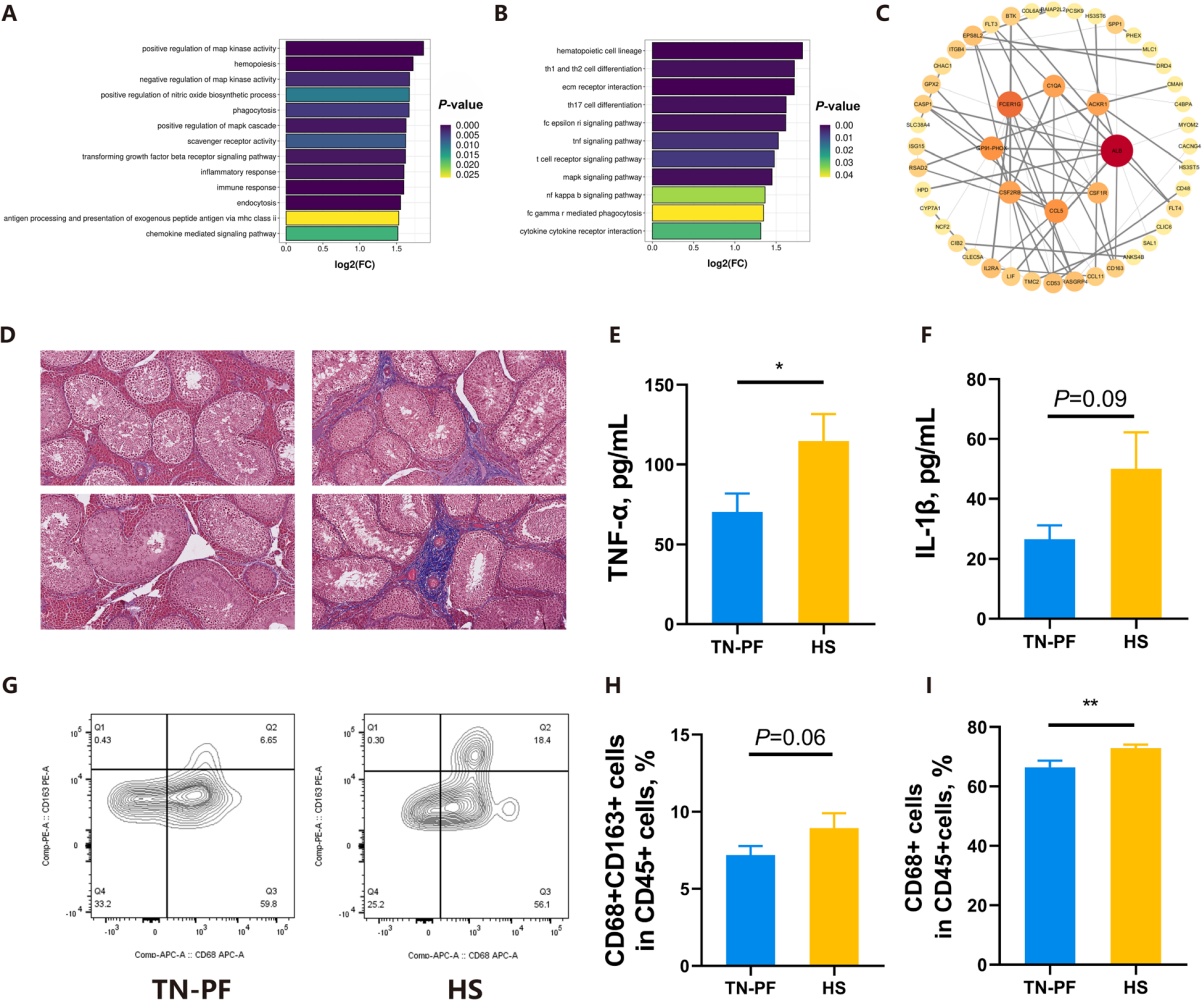
Testicular immunity and orchitis under HS conditions. **A** and** B** GO (A) and KEGG (B) enrichments based on Gene Set Enrichment analysis. **C** Protein-protein interaction network. **D** Representative photos of collagen fibers stained by Masson in the testes. **E** and** F** Levels of TNF-α (E) and IL-1β (F) in plasma isolated from the spermatic vein. **G** Representative flow cytometry quantifications of macrophages in the testes. **H **and** I** Percentages of CD68^+^CD163^+^ and CD68^+^ cells among CD45 ^+^ cells in the testes. ^*^P < 0.05, ^**^P < 0.01

The RNA-seq indicated changes in testicular immunity, such as in the MAPK pathway, T-cell receptor pathways, as well as in TNF-α and hematopoietic cell lineage in KEGG and GO enrichment based on Gene Set Enrichment analysis (Fig. 2A and B). Additionally, a protein–protein interaction network analysis uncovered‌‌ important roles of immunity in testicular changes because genes associated with macrophage and T cells were regarded as key nodes with high degree levels in the network (Fig. 2C). The results were in-line with orchitis, which is characterized by testicular fibrosis, accumulations of inflammatory cytokines, and the invasion of immune cells. In addition, Masson slides exhibited enhanced fibrosis (Fig. 2D) and elevations in TNF-α and IL-1β in spermatic vein plasma were also recorded (*P* < 0.05 and *P* = 0.09, respectively; Fig. 2E and F). An increase in CD68^+^ cells and tendency to have elevated CD68^+^ CD163^+^ cell levels, as observed by flow cytometry, also supported an invasion of immune cells (*P* < 0.01 and *P* = 0.06, respectively; Fig. 2G and H, Fig. S2). Finally, testicular tissue impairment was confirmed by the increased 8-OHG concentration (*P* = 0.05), lowered testosterone concentration (*P* < 0.05), and decreased caspase-3 expression in seminiferous tubules (Fig. S1D–F). These results verified the appearance of testicular impairment and orchitis under HS conditions.

### Changes in complement and engulfment activity in TMs under HS conditions

**Fig. 3:**
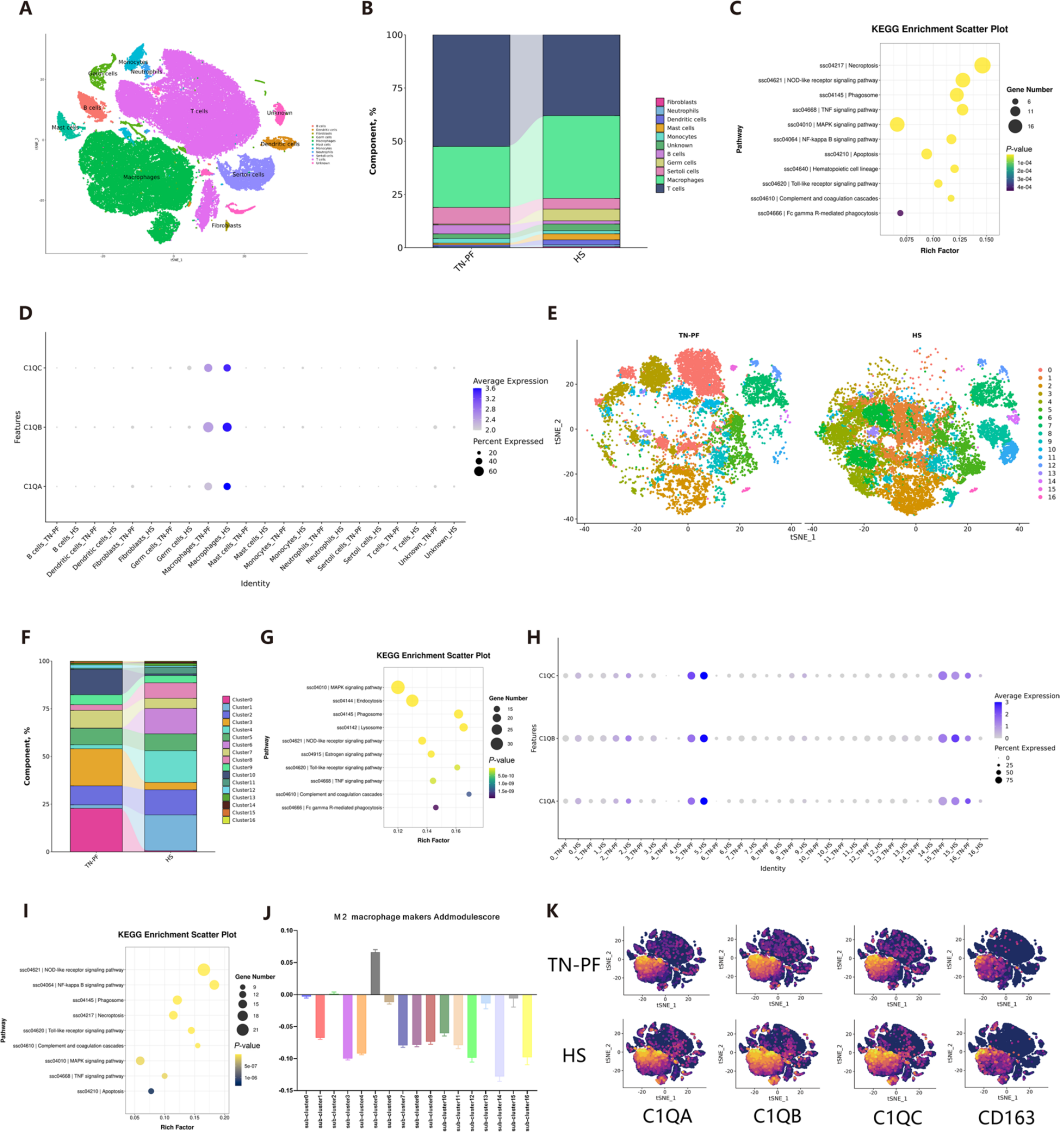
Changes in the TM profile and complement pathway under HS conditions. **A** tSNE graph of identified cells. **B** Percentages of cells in each group. **C** KEGG enrichment of the DEGs from TMs. **D** Expression levels of *C1QA*, *C1QB*, and *C1QC* in testicular cells as assessed by scRNA-seq. **E** tSNE graph of TM subclusters in each group. **F** Percentages of subclusters in each group. **G** KEGG enrichment of marker genes in Subcluster 5. **H** Expression levels of *C1QA*, *C1QB*, and *C1QC* in TM subclusters as assessed by scRNA-seq. **I** KEGG enrichment of DEGs from TM Subcluster 5. **J** Addmodulescore of subclusters. **K** tSNE graph of *C1QA*, *C1QB*, *C1QC*, and *CD163* as assessed by scRNA-seq

For further insights into testicular immunity, flow cytometry sorting was used to purify the testicular CD45^+^ immune cells for scRNA-seq as shown in Fig. S3A, using gating shown in Fig. S3B. A total of 29 clusters of cells with their makers were observed in scRNA-seq (Fig. S3C and D). Most of the clusters were identified based on the cell makers from online databases (Fig. 3A and Fig. S3E). As expected, macrophage presented a large proportion of the testicular immune cells and their DEGs were enriched in cell death, engulfing and inflammatory pathways (Fig. 3B and C). Although we were not able to fully verify cell death and engulfing activity, metabolomics demonstrated the upregulation of lyso-phosphatidylserine 18:1 (*P* < 0.05), phosphatidylserine(18:1(9Z)/0:0) (*P* < 0.05) and lyso-phosphatidylserine 18:2 (*P* < 0.05), which are associated with cell death and engulfment signal, thereby supporting the hypothesis that HS activated engulfment signals in TMs (Fig. S4A–D).

Within the enriched inflammatory pathways, complement and coagulation cascades were noted because complement activation may be responsible for poor male fertility under viral stress [18]. Significant elevations in expression levels of *C1QA*, *C1QB* and *C1QC* both in testes and TMs were confirmed by RNA-seq and scRNA-seq (Fig. 3D, Fig. S4E and F). They also revealed that *C1Q* genes were almost only expressed by TMs (Fig. 3D). The re-clustering of TMs identified 16 sub-clusters, and they showed dramatic shifts in TM subclusters during HS (Fig. 3E and F). In addition, re-clustering revealed Sub-cluster 5 was the exact sub-cluster responsible for C1Q expression, with the KEGG enrichment of Sub-cluster 5 maker genes (Fig. 3G). An expression dotplot showed that Sub-cluster 5 TMs were mainly responsible for C1Q expression, which was accelerated by HS, and KEGG enrichment of DEGs of Sub-cluster 5 also indicated there were changes in complement cascades (Fig. 3H and I).

C1Q activation has been related to CD163^+^ macrophage infiltration of kidney [21]. We next applied an Addmodulescore to identify Sub-cluster 5, which expressed high levels of M2 macrophage genes as determined by comparison to a M2 macrophage gene set from Mossadegh-Keller et al. with slight modifications [22] (Fig. 3J). In agreement with Addmodulescore, a tSNE graph of *C1QA*, *C1QB*, *C1QC* and *CD163* represented a large overlapping area (Fig. 3K). In addition, significant positive linear regression effects were observed between *C1Q* genes and the *CD163* gene or CD68^+^ CD163^+^ cells, corresponding to the TM composition results obtained by flow cytometry (Fig. S4G–L). Collectively, those results indicated the onset of TM engulfment activity and potential in CD163^+^ TM-related complement activation.

### The complement cascades are associated with decrease in semen quality

**Fig. 4:**
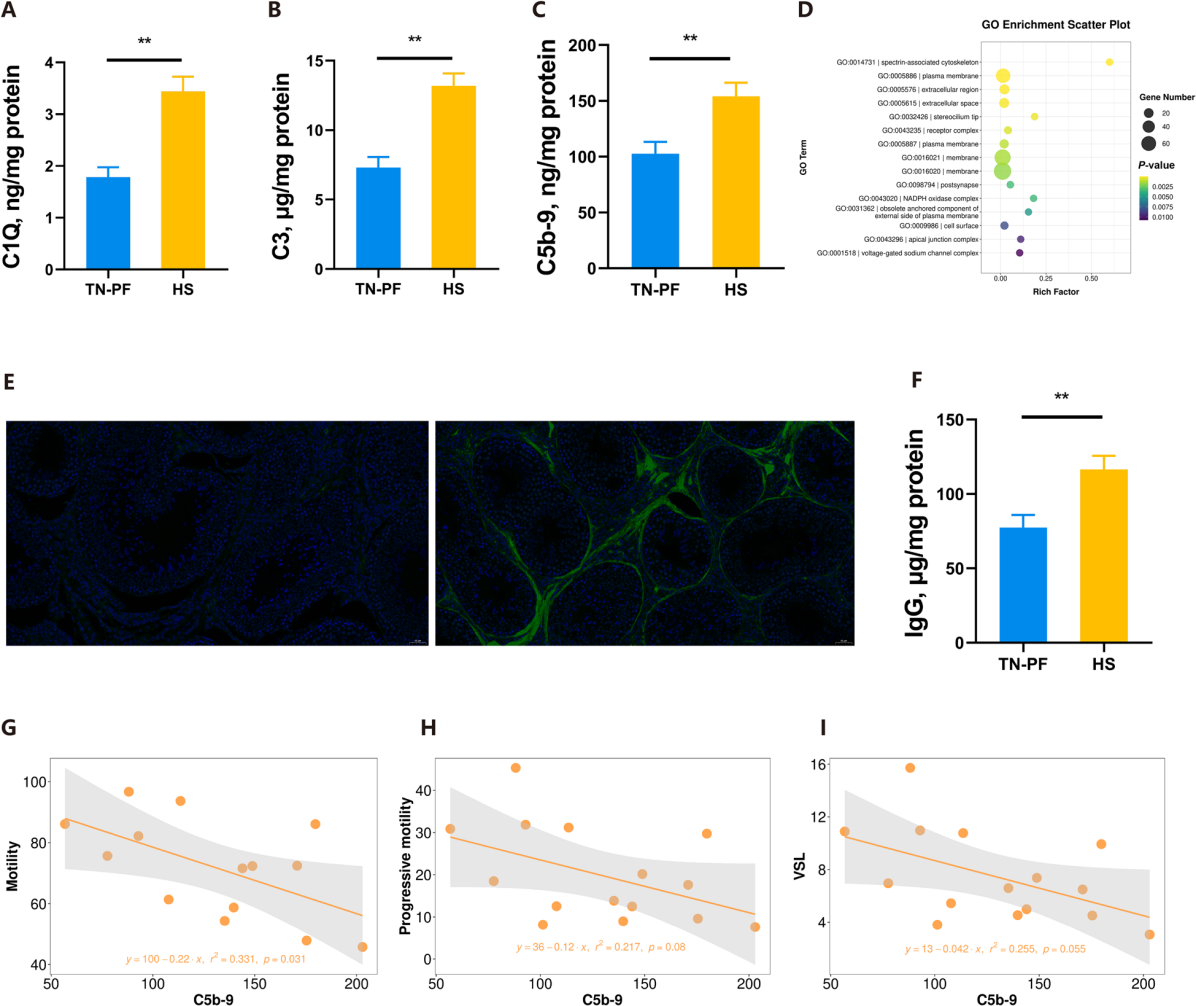
Relationship between complement activation and semen quality. **A–C** Levels of C1Q, C3, and C5b-9 in the testes. **D** GO enrichment of DEGs as assessed by RNA-seq.** E** Level of IgG in testes. **F** Representative photos of IgG immunofluorescence in testes. **G–I** Linear regressions of C5b-9 and sperm motility (**G**), sperm progressive motility (**H**), and sperm VSL (**I**). ^*^P < 0.05, ^**^P < 0.01

**Fig. 5:**
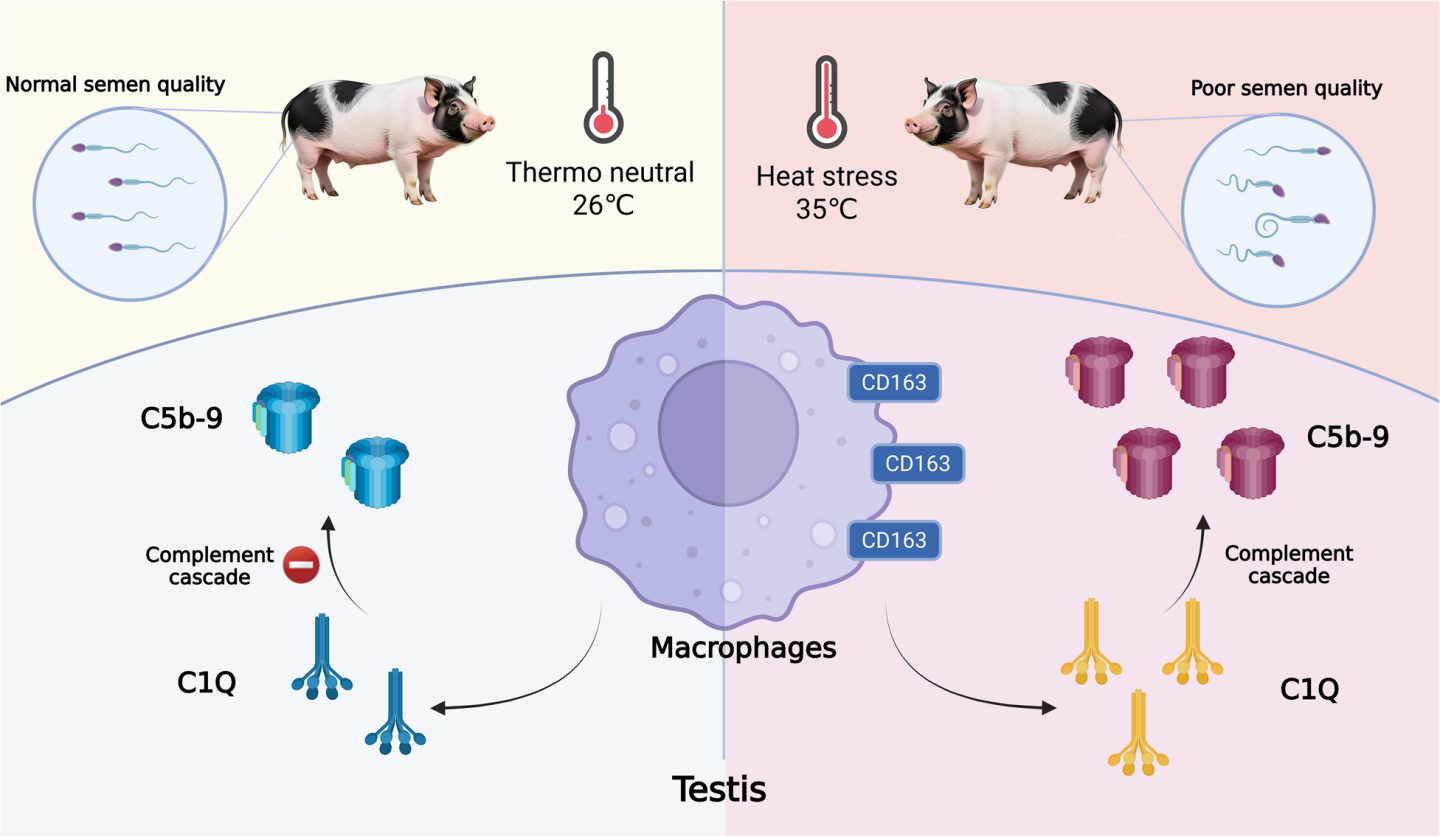
Complement cascades are responsible for the HS-induced poor semen quality in Rongchang boars

C1Q is the key component in the classical activation of complement cascades leading to the final assemble of C5b-9, which specializes in cell membrane breaking and cell lysis, with C3 acting as the key signal transduction component [23]. Unsurprisingly, the C1Q, C3 and C5b-9 proteins were all significantly increased in the testes of heat stressed boars (*P* < 0.01; Fig. 4A–C). The GO enrichment analysis of RNA-seq repeatedly emphasized membrane changes, echoing a key role for complements in testicular changes under HS conditions (Fig. 4D). Because C1Q is key to classical complement activation in which the up-stream activator is an antigen–antibody complex, we visualized the IgG distribution in the testes [18]. As shown in Fig. 4E and F, HS prompted IgG secretion, which potentially accelerated classical complement activation (*P* < 0.01). Most importantly, a linear regression analysis revealed notable, or near-significant, linear correlations between the levels of C5b-9 and semen quality characteristics, at *P* < 0.05 for motility, *P* = 0.08 for progressive motility, and *P* = 0.06 for VSL (Fig. 4G–I). Analogically, C3 and C1Q also exhibited linear associations to semen quality characteristics, with values of *P* = 0.15, *P* = 0.08, *P* = 0.06, and *P* < 0.01 for the motility, progressive motility, VSL, and total abnormality, respectively, for C1Q, and values of *P* = 0.08, *P* = 0.05, *P* < 0.05, and *P* < 0.05 for the motility, progressive motility, VSL, and total abnormality, respectively, for C3 (Fig. S5A–H). Thus, our findings demonstrated a negative association between complement activation and semen quality, emphasizing the role of complement in HS-related decreased semen quality (Fig. 5).

## Discussion

Rongchang boars undergo puberty much earlier than Duroc boars. According to our colleagues in Chongqing, a few motile sperms can be observed in the cauda epididymides of Rongchang boars at the 60 days of age, and appropriate sperm concentration required for our analysis can be acquired after 80 days of age. Consequently, we began the experiment at this specific age.

With global warming, HS has become a tremendous threat. For swine, HS begins at 79 THI and becomes severe at 84 THI [1, 24]. We, therefore, maintained the room temperature at above 35 °C and the THI above 85 to ensure HS reactions in pigs during the HS period. Due to the absence of sweat glands, the RR of pigs increases under HS conditions [25, 26]. In our experiment, an elevation in RR was observed, which was regarded as a hallmark of a HS reaction (*P* < 0.01). Obvious elevations in scrotal temperatures of mice and pigs occur under HS, indicating the possibility of direct impacts of HS on the scrotum [27, 28]. Although scrotal temperature was not strongly altered by HS in our experiment, it was statistically significantly increased in an analysis in which environment temperature was the main effect (*P* < 0.01). We also observed higher HSP60 and HSP90 levels in spermatic vein plasma (*P* < 0.05), which serve as biological confirmation of scrotal HS reactions. The detrimental impact of scrotal HS on male fertility, emphasizing the importance of scrotal temperature for maintaining spermatogenesis, have been determined [6, 8, 29]. The semen quality in our HS group was similarly decreased by lower velocity and motility values and by the increase in sperm abnormalities, which may be regarded as a phenotype of HS boars [2]. In agreement with these findings, a high room temperature of 38 °C for 2 h per day over 14 d lowers the velocity and motility of sperms in mice [4, 30]. Yaeram and colleagues also revealed decreased insemination abilities of sperms from mice subjected to HS of 36 °C for 12 h/d [31]. These data highlight the detrimental effects of HS and elevated scrotal temperature on male reproductivity.

To the best of our knowledge, there is limited information about the connection between HS and orchitis [32]. Although it has been demonstrated that macrophage, as the major testicular immune cell component, was sensitive to hyperthermia characterized by elevated pro-inflammatory and engulfing activities though the NF-kB pathway, TM has been reported to be less pro-inflammatory owing to its poor capacity to activate the NF-kB pathway comparing to peritoneal macrophage [14, 16, 33]. However, in a human model, scrotal HS not only decreases reproductivity but also promotes the level of macrophage migration inhibitory factor, which is responsible for macrophage recruitment and inflammation. This corresponded to the elevation in the TM number we observed [8, 34]. Koch and colleagues also discovered that HS induces the infiltration of macrophages with active inflammatory and phagocytosis responses into the mucosa and submucosa of the jejunum in a bovine model [35]. Similar to their results, our data elucidated an accumulation of inflammatory cytokines due to changes in inflammatory and engulfing pathways in testes, supporting the appearance of HS-induced orchitis.

The orchitis-related testicular impairment could be exerted on Leydig cells, and TNF-α could serve as a histone deacetylase 7 activator. This results in the inhibition of testosterone secretion, which in return blunts the immunosuppressive environment of the testes [12, 36, 37]. The TMs were capable of engulfing early apoptotic Leydig cells, characterized by the release of phosphatidylserine, which corresponded to the increase in phosphatidylserine analogues in our metabolomics analysis. Thus, we hypothesized that there was engulfing of apoptotic Leydig cells under HS conditions, resulting in a reduction in the testosterone level [38]. In addition, both HS and inflammation decrease the integrity of the blood–testis barrier, which is regarded as the guardian of the spermatogenetic environment and required for the isolation of spermatogenetic antigens, resulting in increased inflammation and the leakage of spermatogenetic antigens [39–41]

The complement cascade is rarely focused on during immune response studies, yet it is related to viral stress-induced decreases in male reproductivity [18]. Here, we documented not only accumulations of complement components but also elevated levels of testicular IgG along with HS proteins, identified as self-antigens. These elevations are necessary for the C1Q-related classical activation of the complement cascade, leading to the possibility of antigen–antibody complex formation [18, 42, 43]. While there is little solid evidence regarding the relationship between HS and C1Q, C1Q is often mentioned in anoxic and ischemic models [20, 21, 44]. Coincidentally, HS is associated with the redistribution of blood flow that results in organic ischemic anoxia to facilitate heat ejection [45, 46]. Moreover, C1 inhibitor is capable of alleviating empyrosis-induced tissue impairment [19]. Thus, there are potential reasons to connect HS to the complement reaction and its consequences. Our data verified the onset of C1Q-related complement reactions in testes under HS, yet its impact on semen was only indirectly hypothesized using a linear regression analysis. Thus, further investigations are necessary to fully elucidate the role of the complement cascade in HS-related poor semen quality, perhaps through C1 inhibitor or RNA-interference treatments of animals under HS conditions. Additionally, an applicable strategy to inhibit the complement cascade under industrial conditions should be addressed in the future to facilitate the efficient use of reproductive boars in high-temperature environments.

## Conclusion

Collectively, the results presented support boars suffered from HS-induced orchitis, in which TMs underwent dramatic alterations in engulfment and inflammatory activities. Among the inflammatory pathways, the promotion of C1Q and its down-stream complement cascade by CD163^+^ TMs was associated with poor semen quality through a linear regression analysis.

## Supplementary Information

Additional file 1: Fig. S1. Relationship between HS and testicular impairment. A and B RR (A) and Scrotal temperature (B) under HS conditions. C Scrotal temperatures from 1–5 h after boar were placed in a room at 35 °C. D Representative photos of caspase-3 immunohistochemistry. E and F Levels of 8-OHG (E) and testosterone (F) in plasma isolated from spermatic vein blood.

Additional file 2: Fig. S2. The flow cytometry gating of TMs.

Additional file 3: Fig. S3. Procedures and cell identification in scRNA-seq. A Graphic of the scRNA-seq procedure for isolating CD45^+^ cells. B Gating of flow cytometry sorting. C tSNE graph of automatically defined clusters. D Markers of automatically defined clusters. E Cell markers of cell types.

Additional file 4: Fig. S4. Changes in the testicular profile and the relationship between CD163 and C1Q. A–C Log2 intensities of lyso-phosphatidylserine 18:1 (A), phosphatidylserine (18:1(9Z)/0:0) (B), and lyso-phosphatidylserine 18:2 (C). D KEGG enrichments of significantly altered metabolites. E Expression levels of C1QA, C1QB, and C1QC in testes as assessed by RNA-seq. F KEGG enrichments of DEGs as assessed by RNA-seq. G–L Linear regressions of the expression levels of C1QA and CD163 (G), C1QB and CD163 (H), C1QC and CD163 (I), C1QA and CD68^+ ^CD163^+ ^cells (I), C1QB and CD68^+^ CD163^+^ cells (K), and C1QC and CD68^+^ CD163^+^ cells (L).

Additional file 5: Fig. S5. Linear regressions of semen quality and complement members. A–D Linear regressions of C1Q protein and sperm motility (A), sperm progressive motility (B), sperm VSL (C), and sperm abnormality (D). E–H Linear regressions of C3 protein and sperm motility (E), sperm progressive motility (F), sperm VSL (G), and sperm abnormality (H).

## Data Availability

The raw data of transcriptomics is available at NCBI (Accession: PRJNA1236216), and the raw data of single cell transcriptomics is available at NCBI (Accession: PRJNA1237295), any additional information will be available from the De Wu (wude@sicau.edu.cn) upon request.

## Abbreviations

8-OHG: 8-Hydroxydeoxyguanosine
DEGs: Differentially expressed genes
GO: Gene Ontology
HSP60: Heat shock protein 60
HSP90: Heat shock protein 90
HS: Heat stress
RH: Relative humidity
IL-1β: Interleukin-1β
KEGG: Kyoto Encyclopedia of Genes and Genomes
RR: Respiration rate
TM: Testicular macrophage
THI: Temperature humidity index
TNF-α: Tumor necrosis factor-α
TN-PF: Thermal neutral pair-feed
VSL: Average straight-line velocity
