# Supplementary figures and images for: Heat stress induced testicular impairment is related to orchitis and complement activation in Rongchang boars
Source: J Anim Sci Biotechnol. 2025 Dec 17;16:173. doi: 10.1186/s40104-025-01296-5 (PMC12709842; doi:10.1186/s40104-025-01296-5)

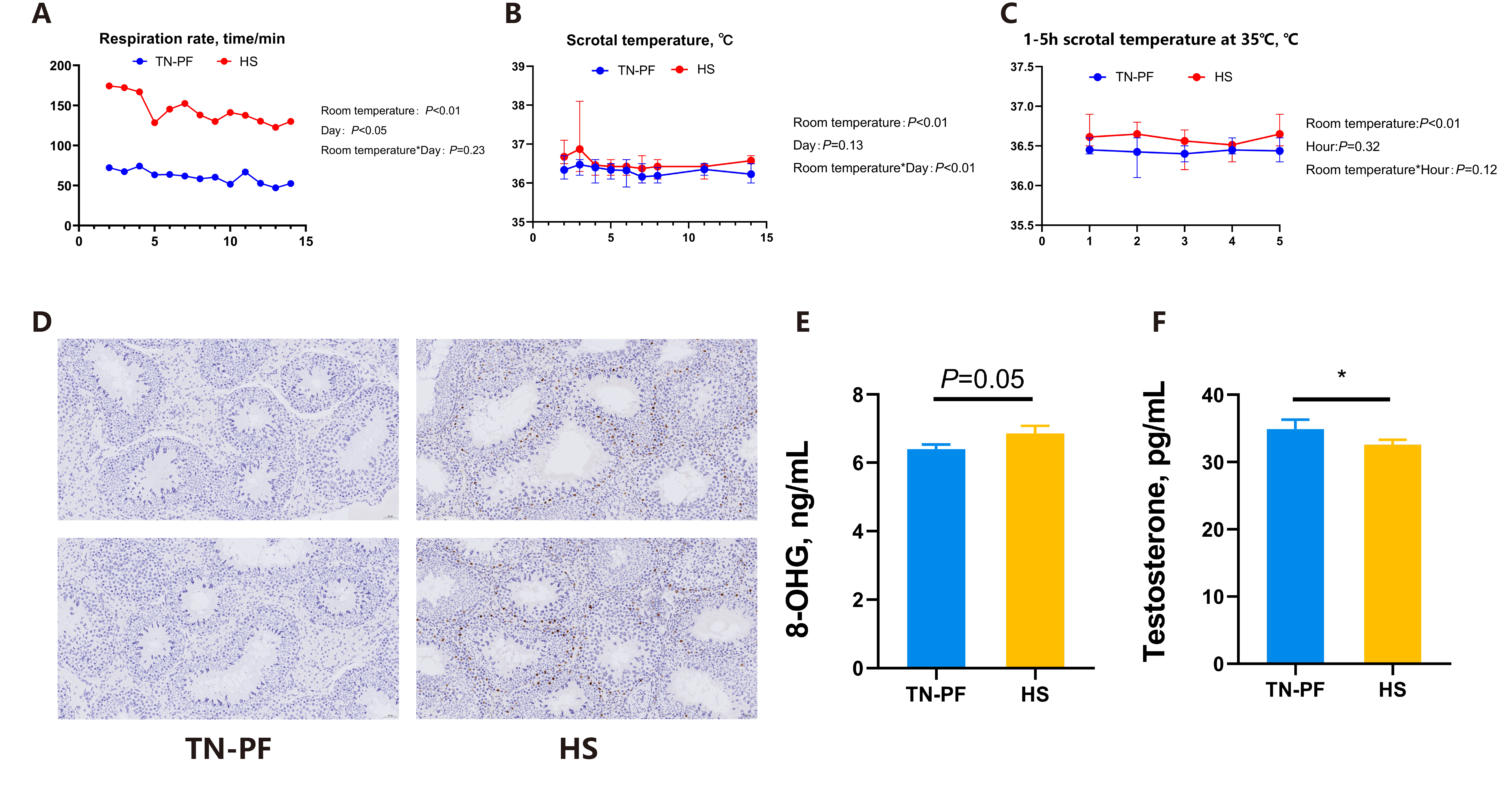

Supplement: Supplementary file 1 — Additional file 1: Fig. S1. Relationship between HS and testicular impairment. A and B RR (A) and Scrotal temperature (B) under HS conditions. C Scrotal temperatures from 1–5 h after boar were placed in a room at 35 °C. D Representative photos of caspase-3 immunohistochemistry. E and F Levels of 8-OHG (E) and testosterone (F) in plasma isolated from spermatic vein blood. [file 40104_2025_1296_MOESM1_ESM.png]

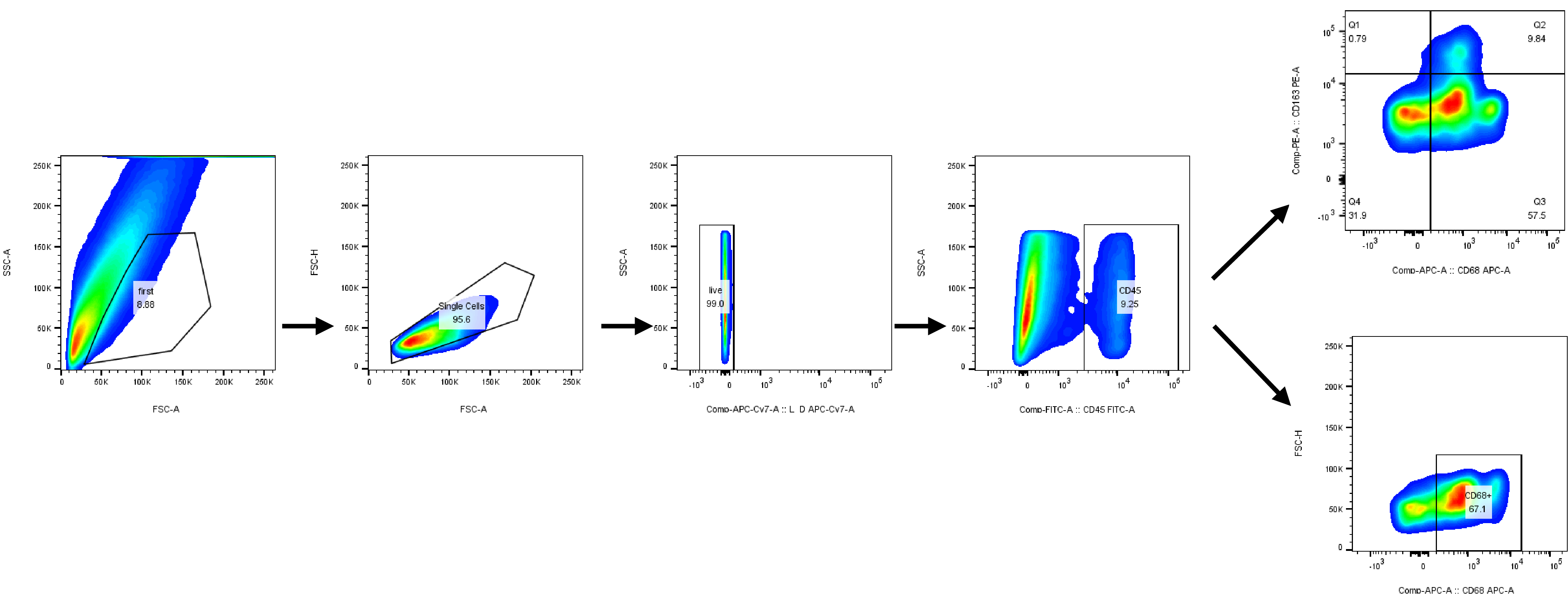

Supplement: Supplementary file 2 — Additional file 2: Fig. S2. The flow cytometry gating of TMs. [file 40104_2025_1296_MOESM2_ESM.png]

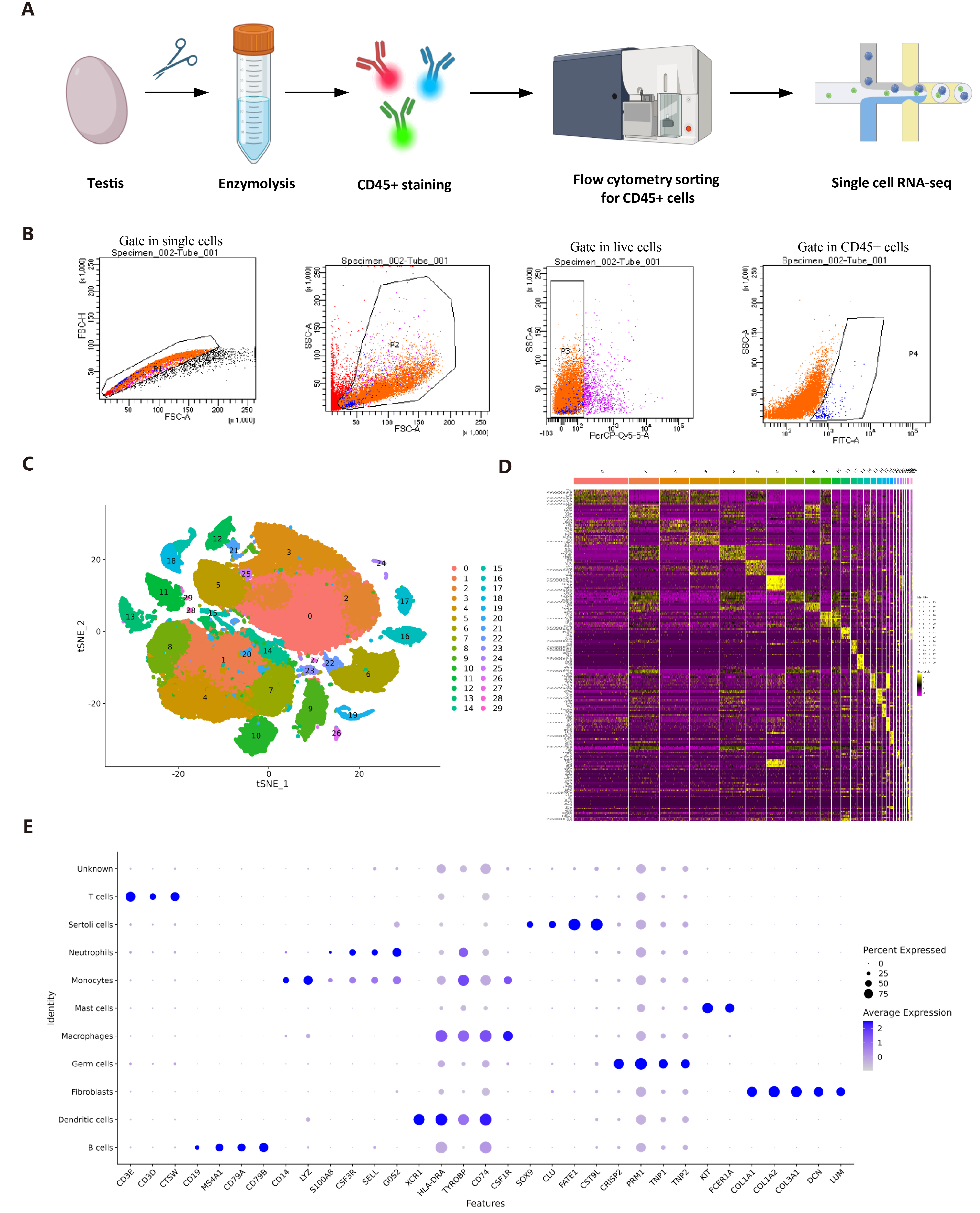

Supplement: Supplementary file 3 — Additional file 3: Fig. S3. Procedures and cell identification in scRNA-seq. A Graphic of the scRNA-seq procedure for isolating CD45+ cells. B Gating of flow cytometry sorting. C tSNE graph of automatically defined clusters. D Markers of automatically defined clusters. E Cell markers of cell types. [file 40104_2025_1296_MOESM3_ESM.png]

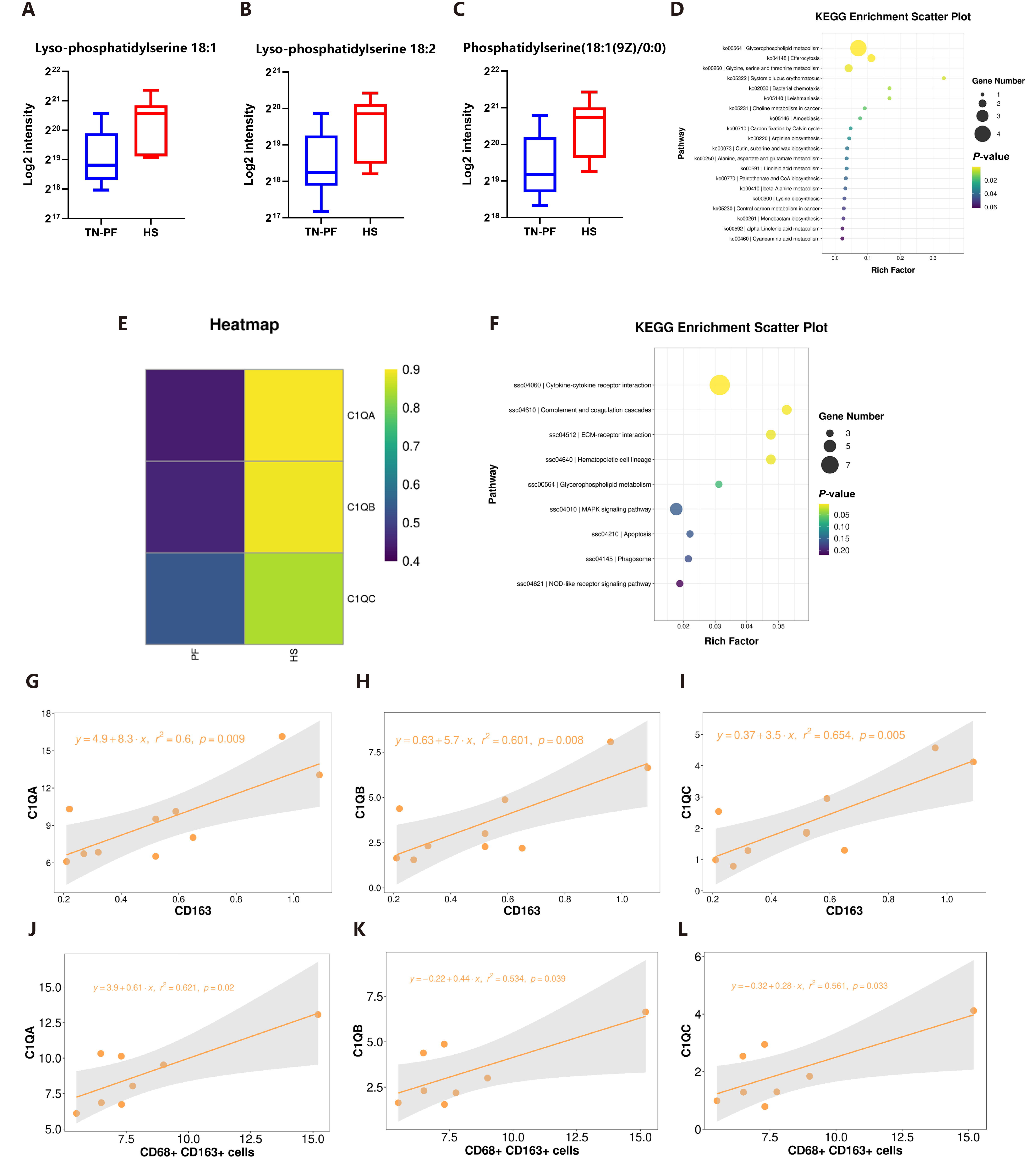

Supplement: Supplementary file 4 — Additional file 4: Fig. S4. Changes in the testicular profile and the relationship between CD163 and C1Q. A–C Log2 intensities of lyso-phosphatidylserine 18:1 (A), phosphatidylserine (18:1(9Z)/0:0) (B), and lyso-phosphatidylserine 18:2 (C). D KEGG enrichments of significantly altered metabolites. E Expression levels of C1QA, C1QB, and C1QC in testes as assessed by RNA-seq. F KEGG enrichments of DEGs as assessed by RNA-seq. G–L Linear regressions of the expression levels of C1QA and CD163 (G), C1QB and CD163 (H), C1QC and CD163 (I), C1QA and CD68+ CD163+ cells (I), C1QB and CD68+ CD163+ cells (K), and C1QC and CD68+ CD163+ cells (L). [file 40104_2025_1296_MOESM4_ESM.png]

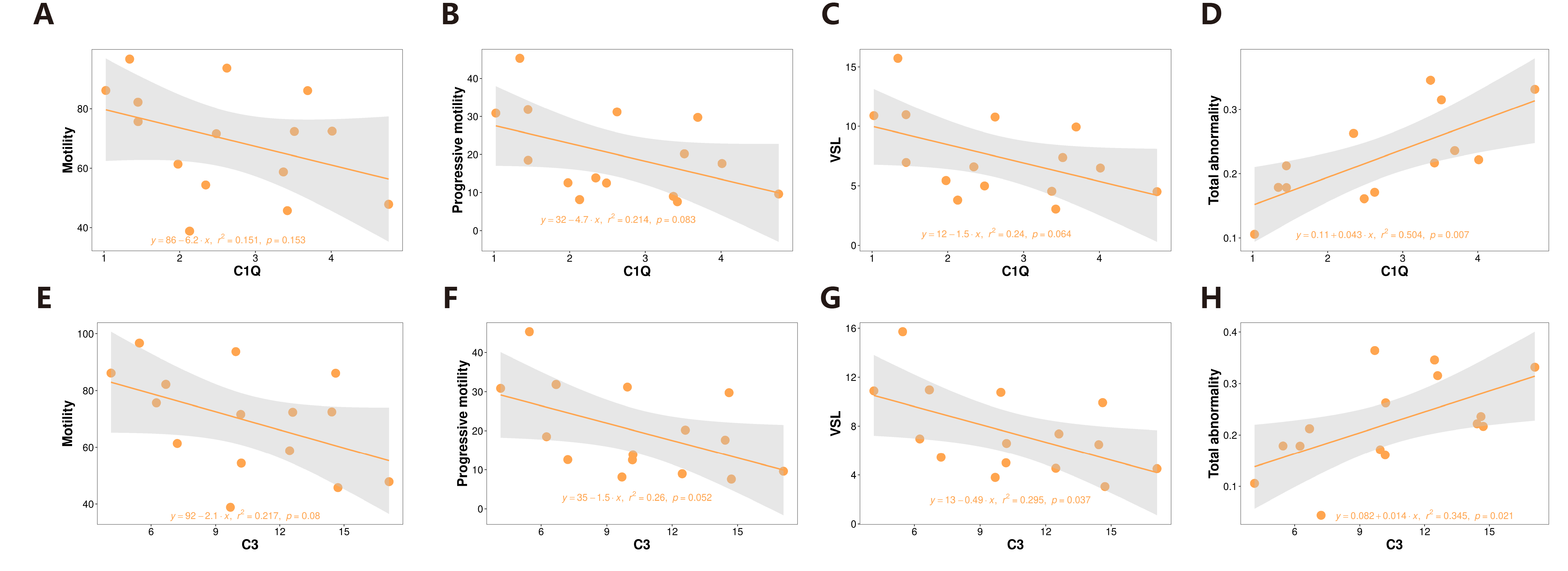

Supplement: Supplementary file 5 — Additional file 5: Fig. S5. Linear regressions of semen quality and complement members. A–D Linear regressions of C1Q protein and sperm motility (A), sperm progressive motility (B), sperm VSL (C), and sperm abnormality (D). E–H Linear regressions of C3 protein and sperm motility (E), sperm progressive motility (F), sperm VSL (G), and sperm abnormality (H). [file 40104_2025_1296_MOESM5_ESM.png]
